# Evaluation of Pneumonia Severity and Acute Physiology Scores to Predict ICU Admission and Mortality in Patients Hospitalized for Influenza

**DOI:** 10.1371/journal.pone.0009563

**Published:** 2010-03-05

**Authors:** Matthew P. Muller, Allison J. McGeer, Kazi Hassan, John Marshall, Michael Christian, for the Toronto Invasive Bacterial Disease Network

**Affiliations:** 1 Department of Medicine, St. Michael's Hospital, University of Toronto, Toronto, Canada; 2 Department of Laboratory Medicine and Pathobiology, Mount Sinai Hospital, University of Toronto, Toronto, Canada; 3 Mount Sinai Hospital, University of Toronto, Toronto, Canada; 4 Department of Surgery, St. Michael's Hospital, University of Toronto, Toronto, Canada; 5 Department of Medicine, Mount Sinai Hospital and University Health Network, University of Toronto, Toronto, Canada; 6 Canadian Forces Health Services, Toronto, Canada; Duke University, United States of America

## Abstract

**Background:**

The demand for inpatient medical services increases during influenza season. A scoring system capable of identifying influenza patients at low risk death or ICU admission could help clinicians make hospital admission decisions.

**Methods:**

Hospitalized patients with laboratory confirmed influenza were identified over 3 influenza seasons at 25 Ontario hospitals. Each patient was assigned a score for 6 pneumonia severity and 2 sepsis scores using the first data available following their registration in the emergency room. In-hospital mortality and ICU admission were the outcomes. Score performance was assessed using the area under the receiver operating characteristic curve (AUC) and the sensitivity and specificity for identifying low risk patients (risk of outcome <5%).

**Results:**

The cohort consisted of 607 adult patients. Mean age was 76 years, 12% of patients died (71/607) and 9% required ICU care (55/607). None of the scores examined demonstrated good discriminatory ability (AUC≥0.80). The Pneumonia Severity Index (AUC 0.78, 95% CI 0.72–0.83) and the Mortality in Emergency Department Sepsis score (AUC 0.77, 95% 0.71–0.83) demonstrated fair predictive ability (AUC≥0.70) for in-hospital mortality. The best predictor of ICU admission was SMART-COP (AUC 0.73, 95% CI 0.67–0.79). All other scores were poor predictors (AUC <0.70) of either outcome. If patients classified as low risk for in-hospital mortality using the PSI were discharged, 35% of admissions would have been avoided.

**Conclusions:**

None of the scores studied were good predictors of in-hospital mortality or ICU admission. The PSI and MEDS score were fair predictors of death and if these results are validated, their use could reduce influenza admission rates significantly.

## Introduction

Seasonal influenza results in over 50,000 deaths and 500,000 hospitalizations per year in the US alone [1], [2]. Emergency departments (ER) and inpatient medical services often operate near maximum capacity during a moderately severe influenza season [3]. The current H1N1 influenza pandemic is further overloading healthcare systems as it spreads globally though populations with minimal immunity to this virus.

Thus, in a busy influenza season or during a pandemic, clinicians need to be able to rapidly assess large numbers of patients with influenza-like illness (ILI) and identify those patients that require inpatient care. The inappropriate admission of low risk patients and the failure to admit high risk patients could lead to widespread inefficiency within the healthcare system, and poor outcomes for some patients.

An influenza severity score designed for use at the time that admission decisions are made and capable of discriminating between patients at high and low risk of severe disease and death would be a valuable tool that could be used to develop clinical pathways or decision support tools for use during influenza season or during a pandemic, similar to those used for community acquired pneumonia. Pneumonia severity scores such as the Pneumonia Severity Index (PSI) [1], [4] and the CURB-65 score [5], [6] have already been developed and are widely used to support admission decisions for patients with community-acquired pneumonia (CAP). The CURB-65, the SOFA score, the Simple Triage Scoring System (STSS) and the Pandemic Medical Early Warning Score (PMEWS) have all been recommended for use during a pandemic [3], [7]–[11]. Unfortunately, none of these scores have been evaluated using patients with influenza. To address this issue, we assessed the performance of 13 pneumonia severity and acute physiology scores in a population of patients hospitalized with influenza.

## Methods

### Ethics

Patients were identified by the microbiology laboratories at all participating hospitals. Identified patients were approached and written informed consent was obtained for inclusion in the study and for chart review. Ethics approval for this study was obtained from the research ethics boards of all participating hospitals.

### Setting and Study Population

This study was conducted by the Toronto Invasive Bacterial Disease Network (TIBDN). TIBDN is a network of 25 hospitals that conduct population-based surveillance for infectious diseases in the Metropolitan Toronto and Peel Region, Ontario (population 3.7 million). Since January 1, 2005 TIBDN hospitals have been conducting active surveillance for patients admitted to hospital with laboratory confirmed influenza. Details of the surveillance methodology have been previously published [12].

We included in our cohort all adult (age≥15 years) patients admitted to a TIBDN hospital because of disease associated with laboratory confirmed influenza during the 2005, 2006 or 2007 influenza season (January 2005 to April 2007). Patients were considered to have laboratory confirmed influenza if any specimen tested positive for influenza A or B by enzyme immunoassay, viral culture or PCR [12].

### Prognostic Scoring Systems

Scoring systems with potential usefulness in evaluating patients with seasonal or pandemic influenza at the time of initial ER assessment were identified through a MEDLINE search and expert consultation (Table 1). Scores were considered if they were designed for use at the time of ER assessment and/or admission, were derived or validated in a cohort of patients with influenza, pneumonia or sepsis, and were intended to predict relevant outcomes (i.e. death, mechanical ventilation, ICU admission).

**Table 1:** Prognostic Scoring Systems.

Scoring System	Patient Population	Abbreviation
CURB-65 [21]	CAP	CURB65
Mortality in Emergency Department Sepsis [20]	Sepsis	MEDS
Nursing Home Acquired Pneumonia in the Elderly [22]	CAP	NHAP
Pandemic Medical Early Warning Score [7]	CAP	PMEWS
Pneumonia Severity Index [23]	CAP	PSI
Severity Score for the Elderly with Community Acquired Pneumonia [24]	CAP	CAP
SMART-COP [25]	CAP	SMARTCOP
Simple Triage Scoring System [11]	Sepsis	STSS

### Study Outcomes

The primary outcome for this study was in-hospital mortality. The secondary outcome was admission to ICU.

### Data Collection

Trained data abstractors used a standardized data collection form to collect information on patient outcomes and all of the demographic, clinical, laboratory and radiographic data required to calculate each score. Only records obtained within 24 hours of registration in the ER were included, and if >1 result was obtained, the first recorded value was used to calculate the score. Missing data was assumed to be normal. Data were entered in duplicate, cleaned and analyzed in SAS (SAS version 9.0, SAS Institute, Cary NC).

### Statistical Methods

All prognostic scores were calculated for each patient using the first data available after their arrival in the ER. The accuracy of each score in predicting outcome was evaluated using measures of discrimination (how well each score discriminates between patients that do or do not progress to the outcome) and calibration (whether the probabilities predicted by the score are, on average, close to the observed outcomes) [13].

Discrimination was assessed by plotting the receiver operating characteristic (ROC) curve and calculating the area under the ROC curve (AUC) [14], [15]. The AUC is a standard method of measuring and comparing the discrimination of multiple predictive models. AUC values were ‘ranked’ as excellent (AUC≥0.90), good (AUC≥0.80 and <0.90), fair (AUC≥0.70 and <0.80) and poor (<0.70). Confidence intervals around the AUC were calculated using bootstrap re-sampling methods with 1000 repetitions [16]. Additionally, the sensitivity and specificity of each score were calculated by using as a cutoff the value of each score that separated patients with a predicted outcome risk of <5% from those at higher risk.

Calibration was assessed graphically using quintile plots of observed versus expected outcome and statistically using the Hosmer-Lemeshow C statistic [17]. The Hosmer-Lemeshow C statistic compares observed vs. expected outcome within deciles of the expected outcome. A statistically significant result suggests a lack of calibration.

For all analyses, predictor variables with missing data were assumed to be normal. To validate this approach, we compared outcomes between patients with and without missing data and repeated the analysis using multiple imputation to impute missing values for all variables [18].

## Results

### Missing Data

Outcome data were complete. Data on predictor variables were nearly complete (<4% missing) for all variables except arterial blood gases, albumin and blood urea nitrogen which were missing in 70%, 68% and 11% of cases. For these variables, the case fatality rate was the same or lower in patients with missing data compared to patients with documented results, supporting our assumption that missing variables were likely within the normal range [data not shown].

### Cohort Description

We identified 617 hospitalized adult patients with laboratory confirmed influenza over 3 influenza seasons. Of these, 10 were not included either due to lack of informed consent (n = 5) or lack of access to their medical records (n = 5). Therefore, 607 patients (98%) were included. The median (IQR) age was 76 years (64 years to 83 years), 49% of cases were female and >80% of patients had one or more chronic medical illness. Patients were admitted directly from home (83%), from nursing homes (13%), or from other chronic care facilities (4%) (Table 2).

**Table 2:** Demographic and clinical characteristics of patients with laboratory confirmed influenza in TIBDN hospitals, 2005–2007.

	Total (n = 607)	Survived (n = 536)	Died (n = 71)
**Demographics**			
Age (years, IQR)	76 (64–83)	76 (62–82)	78 (72–85)
Female	49% (297/607)	49% (264/536)	46% (33/71)
Residence prior to hospitalization			
Community	83% (506/607)	85% (453/536)	75% (53/71)
Nursing Home	13% (76/607)	12% (63/536)	18% (13/71)
Other Institutional	4% (25/607)	4% (20/536)	7% (5/71)
**Comorbidities and other risk factors for severe influenza**			
Age>65	74% (451/607)	73% (390/536)	86% (61/71)
Pregnancy	2% (10/607)	2% (10/536)	0% (0/71)
Any comorbid illness	88% (536/607)	87% (468/536)	96% (68/71)
Respiratory (including asthma)	42% (257/607)	42% (226/536)	44% (31/71)
Cardiac (excluding hypertension)	59% (360/607)	58% (309/536)	72% (51/71)
Immunosuppression (including diabetes)	61% (372/607)	60% (323/536)	69% (49/71)
Neuromuscular disorder	8% (47/607)	6% (33/536)	20% (14/71)
Any influenza risk factor	94% (568/607)	93% (493/536)	99% (70/71)
**Clinical Features**			
Fever	85% (517/607)	86% (461/536)	79% (56/71)
Cough	90% (546/607)	91% (487/536)	82% (58/71)
Dyspnea	63% (384/607)	61% (326/536)	82% (58/71)
Fatigue	61% (372/607)	61% (329/536)	61% (43/71)
Temperature >38.0°C	48% (291/607)	50% (266/536)	35% (25/71)
Heart Rate >120 beats/minute	13% (79/607)	13% (67/536)	17% (12/71)
Respiratory Rate >30 breaths/minute	9% (55/607)	9% (46/536)	13% (9/536)
Hypotension (Systolic blood pressure <90 mmHg)	5% (31/607)	5% (25/536)	8% (6/71)
**Laboratory ** **Results** ** and Radiography**			
White blood cell count (mean, SD)	9.6 (7.6)	9.2 (7.3)	12.6 (8.5)
Platelet count (mean, SD)	216 (90)	214 (88)	224 (106)
Hematocrit (mean, SD)	0.38 (0.06)	0.38 (0.06)	0.37 (0.07)
Sodium (mean, SD)	126 (9)	136 (9)	138 (9)
Creatinine (mean, SD)	122 (111)	119 (107)	146 (136)
Albumin (mean, SD)	32 (7)	33 (7)	29 (7)
AST (mean, SD)	64 (158)	47 (71)	159 (365)
Bilirubin (mean, SD)	17 (46)	17 (49)	16 (19)
Infiltrate on initial Chest X-ray	47% (273/583)	45% (232/514)	59% (41/69)
**Treatment**			
Antiviral Use	40% (245/607)	41% (222/536)	32% (23/71)

*influenza risk factors include age >65, pregnancy, and comorbid conditions including chronic cardiorespiratory illness, immunosuppressive illness and neuromuscular illness.

The most common presenting symptoms were cough (90%), fever (68%), shortness of breath (63%) and fatigue (61%). Chest x-ray evidence consistent with pneumonia was seen on the initial chest x-ray in 45% of cases (Table 2).

Overall, 16% (n = 96) of patients required ICU admission, 9% (n = 55) required mechanical ventilation, and 12% (n = 71) died. The median time (IQR) to ICU admission for patients that required ICU was 0 days (0 days to 2 days) and the median duration of ICU stay was 6 days (4 days to 14 days). The median time (IQR) to mechanical ventilation among those requiring ventilation was 0 days (0 days to 4 days) and the median duration of ventilation was 5 days (2 days to 13 days). The median time to death among fatal cases (IQR) was 13 days (3 days to 25 days).

### Performance of Scores for the Prediction of Death

**Figure 1:**
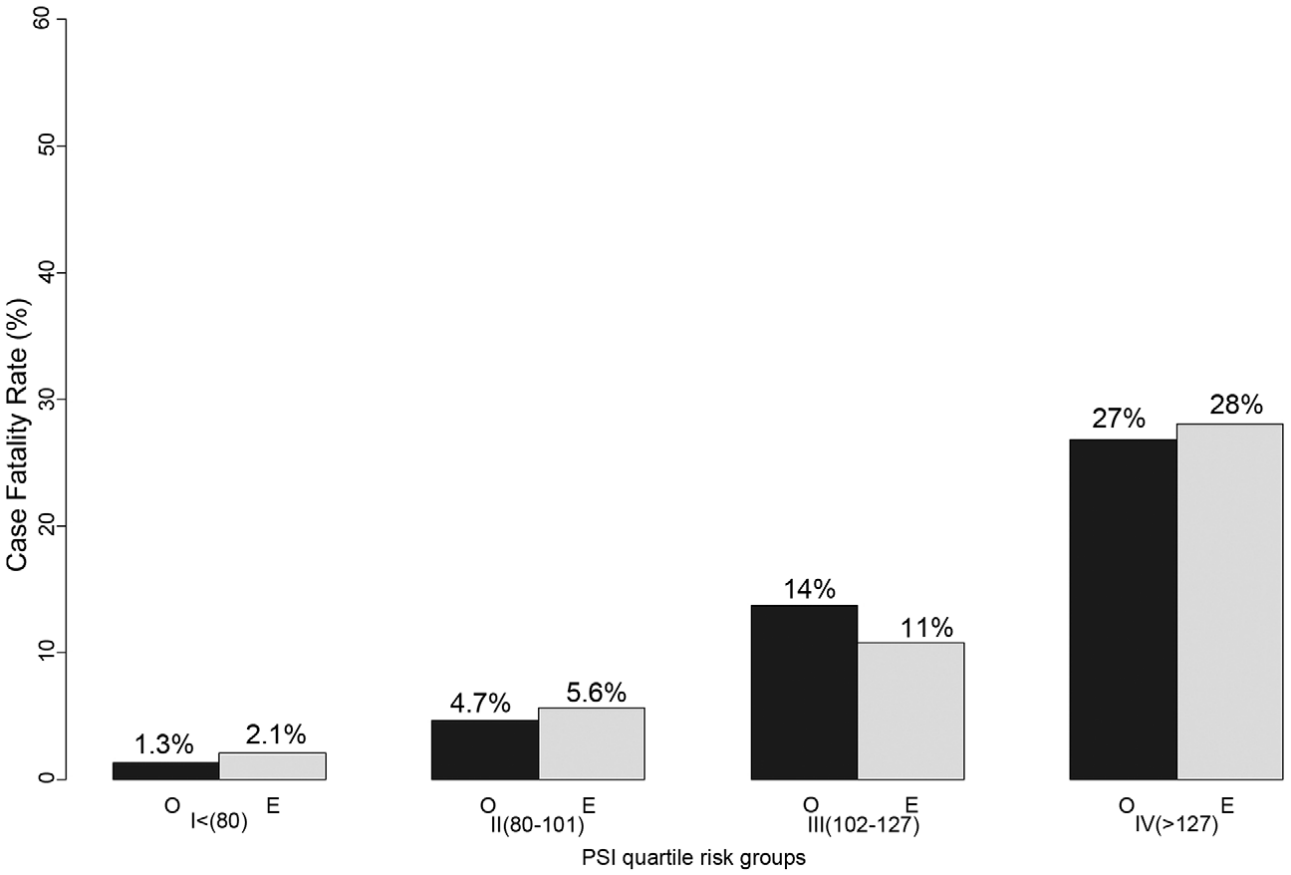
PSI score: observed and predicted mortality in quartile groups. Observed (black) and predicted (grey) in-hospital mortality are similar within quartiles defined by the PSI score. In the lowest quartile (patients with PSI<80), in-hospital mortality was low (<5%).

**Figure 2:**
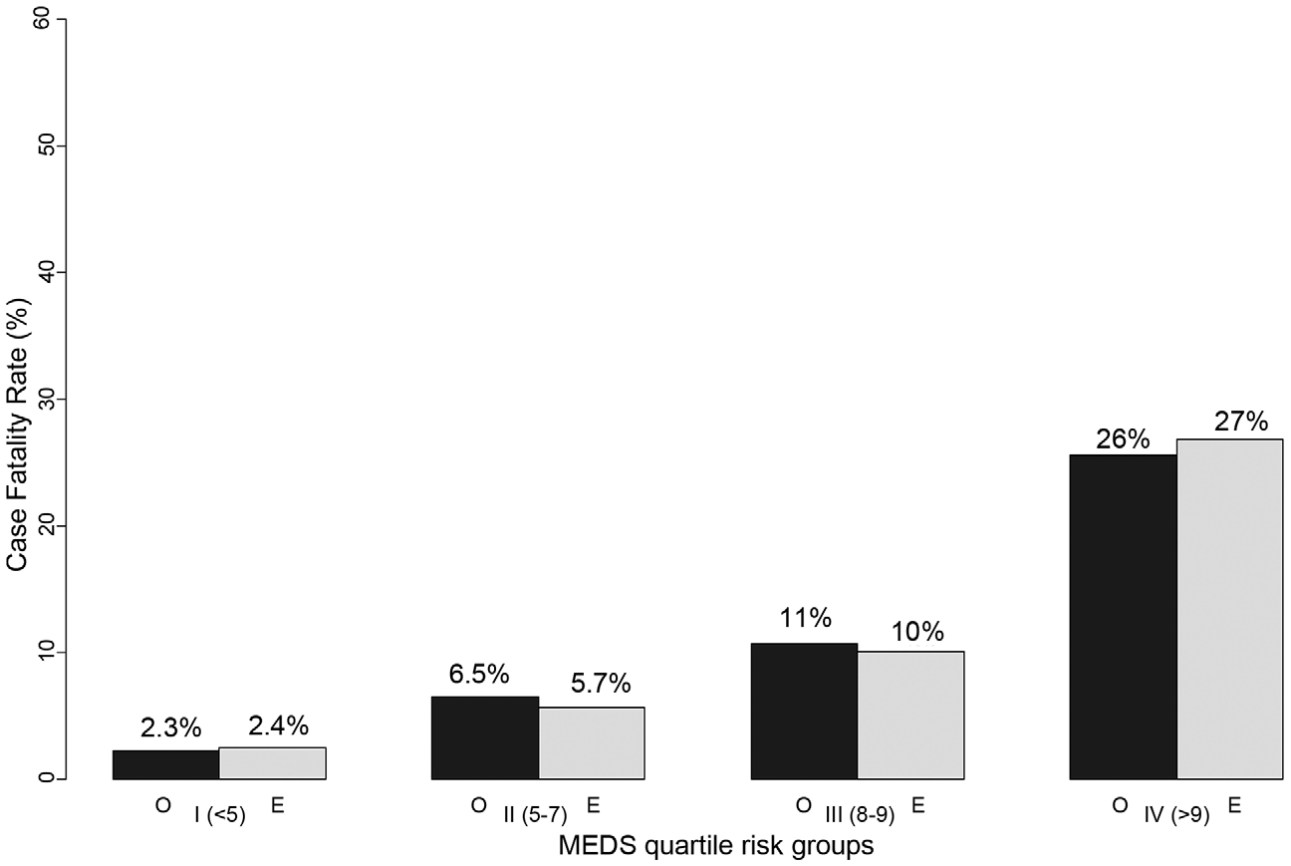
MEDS score: observed and predicted mortality in quartile groups. Observed (black) and predicted (grey) in-hospital mortality are similar within quartiles defined by the PSI. In the lowest quartile (patients with PSI<80), in-hospital mortality was low (<5%).

The AUC for the prediction of death ranged from 0.78 to 0.65 (Table 3). The two best predictors of in-hospital mortality were the PSI (AUC = 0.79, 95% CI 0.72–0.83) and the MEDS (AUC = 0.78, 95% CI 0.71 to 0.83). These were the only with confidence intervals including AUC >0.80 (Table 3). All of the scores were well calibrated with the exception of the NHAP which demonstrated statistically significant lack of fit (Table 3). A graphical depiction of the correlation between observed and predicted in-hospital mortality for the top two performing scores is shown in Figure 1 and 2. When the analysis was repeated using multiple imputation to replace missing data, the order and magnitude of the observed AUC values were unchanged [data not shown].

**Table 3:** AUC and Hosmer Lemeshow Goodness of Fit Test for the prediction of in-hospital mortality.

Score	Death* AUC (95% CI)	Hosmer-Lemeshow C statistic(Chi-Square)*
PSI	0.78 (0.72–0.83)	3.9
MEDS	0.77 (0.71–0.83)	12.6
STSS	0.71 (0.66–0.77)	1.4
SMARTCOP	0.69 (0.62–0.75)	4.3
PMEWS	0.68 (0.61–0.74)	7.4
NHAP	0.68 (0.62–0.74)	10.6*
CURB65	0.66 (0.60–0.72)	4.8
CAP	0.65 (0.58–0.71)	3.3

*p<0.05.

When these scores were used to identify a subset of patients at low risk of death (<5% risk of death) who could potentially be managed as outpatients, the sensitivities of the scores for identifying patients at risk of in-hospital mortality were good (≥85% for all scores) but the specificity was poor (≤ 40% for all scores) (Table 4). The sensitivity and specificity for the PSI score were 93% and 39%. Using the PSI score to guide admission decisions (i.e. admitting only patients with a PSI score >87 and an estimated risk of in-hospital mortality of ≥5%) would have reduced admissions by 35%. The case fatality rate in patients with scores of ≤ 87 was 5/231 (2.3%). The sensitivity and specificity of the MEDS score were 85% and 40%. Using the MEDS score to guide admission decisions (i.e. admitting only patients with a MEDS score >5 and an estimated risk of in-hospital mortality of ≥5%) would have reduced admissions by 38%. The case fatality rate in patients with a score ≤ 5 was 11/228 (4.8%).

**Table 4:** Sensitivity and Specificity of scores for the identification of patients at low risk of death (predicted probability death <5%).

Score	Sensitivity	Specificity	Reduction in Admission	Fatal cases sent home (false negatives)
PSI	93%	39%	35%	5/213 (2.3%)
MEDS	85%	40%	38%	11/228 (4.8%)
STSS	99%	15%	14%	1/84 (1.1%)
SMARTCOP	100%	2.8%	2.5%	0/15 (0%)
PMEWS	97%	13%	12%	2/74 (2.7%)
CURB65	97%	16%	14%	2/87 (2.2%)

*NHAP and CAP did not classify any patient as having a <5% risk of death.

### Performance of Scores in the Prediction of ICU Admission

**Figure 3:**
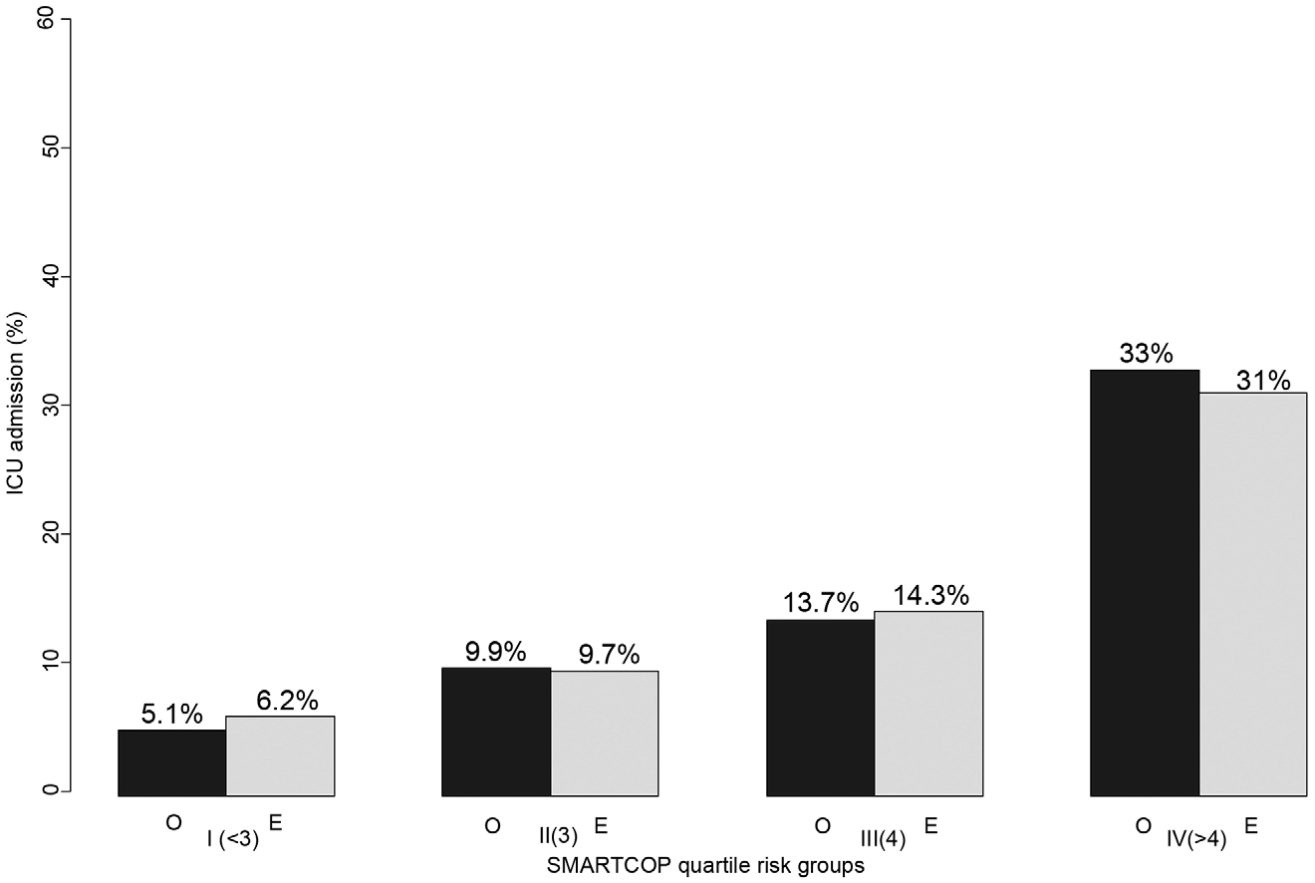
SMARTCOP: observed and predicted ICU admission in quartile groups. Observed (black) and predicted (grey) ICU admission are similar within quartiles defined by SMARTCOP. In the quartile (patients with SMARTCOP<3), the incidence of ICU admission was low (<5%).

The AUC for the prediction of ICU admission ranged from 0.73 to 0.58 (Table 5). The best predictor of ICU admission was SMARTCOP (AUC = 0.73, 95% CI 0.67–0.79). No score demonstrated good performance (AUC≥0.80) or included an AUC = 0.80 within the 95% confidence intervals. Furthermore, SMARTCOP was not well calibrated, and demonstrated a statistically significant lack of fit (Table 5). A graphical depiction of the correlation between observed and predicted ICU admission for SMARTCOP is shown in figure 3. When the analysis was repeated using multiple imputation to replace missing data, the order and magnitude of the observed AUC values were unchanged [data not shown].

**Table 5:** AUC and Hosmer Lemeshow Goodness of Fit Test for the prediction of the need for ICU admission.

Score	ICU Admission AUC (95% CI)	Hosmer-Lemeshow C statistic (Chi-Square)
SMARTCOP	0.73 (0.67–0.79)	33.0*
MEDS	0.67 (0.61–0.73)	11.7
PSI	0.67 (0.61–0.73)	11.1
CAP	0.65 (0.59–0.71)	0.92
PMEWS	0.63 (0.57–0.69)	12.0
STSS	0.63 (0.57–0.69)	2.1
NHAP	0.62 (0.57–0.68)	3.2
CURB65	0.58 (0.52–0.64)	2.4

*p<0.05.

When these scores were used to identify a subset of patients at low risk of requiring ICU care (<5% risk of admission to ICU) who could potentially be managed as outpatients, most scores failed to identify any patients within this low risk category (i.e. the estimated risk of ICU admission was >5% for all patients). Only the PSI and SMARTCOP scores identified a subset of patients at low risk. The sensitivity of both these scores for detecting patients at risk of ICU admission was 100% (i.e. no patient classified as low risk required ICU care) but the specificities were both poor (6.0% for PSI and 2.8% for SMARTCOP) and as a result, neither score would likely be useful in reducing the number of hospital admissions (e.g. admitting only patients with a SMARTCOP score >1 and an estimated risk of ICU admission of ≥5% would reduce admissions by only 2.5%) (Table 6).

**Table 6:** Sensitivity and Specificity of scores for the identification of patients at low risk of ICU admission (predicted probability ICU admission <5%).

Score	Sensitivity	Specificity	Reduction in Admission	Fatal cases sent home
PSI	100%	6.0%	5.2%	0/32 (0%)
SMARTCOP	100%	2.8%	2.5%	0/15 (0%)

*MEDS, STSS, PMEWS, CURB65, NHAP and CAP did not classify any patient as having a <5% risk of death.

### Re-Examination of Data with Fatal Cases Not Admitted to ICU Excluded

Because we did not record the ‘do not resuscitate’ status of patients in our cohort, we conducted a post-hoc analysis with patients that died outside of the ICU setting excluded as this group may have included ‘do not resuscitate’ patients that would otherwise have prognostic features predictive of the need for ICU admission (e.g. severe hypoxemia) and may have reduced the ability of scores to predict ICU admission accurately.

In this post-hoc analysis, 36/71 fatal cases were excluded. When we re-analyze our data after excluding patients that died outside of the ICU, there was little difference in the results, with small increases in AUC seen for most scores (Tables 7, 8). The only exception was for SMARTCOP, which demonstrated a substantial improvement in its ability to predict in-hospital death (AUC 0.79, 95% CI 0.73 to 0.86) while remaining the best predictor of ICU admission (AUC = 0.73, 95% CI 0.68 to 0.79).

**Table 7:** Prediction of in-hospital mortality with fatal cases not admitted to ICU excluded.

Score	Death* AUC (95% CI)	Hosmer-Lemeshow C statistic (Chi-Square)*
SMARTCOP	0.79 (0.73–0.85)	5.3
MEDS	0.79 (0.69–0.87)	13.7
PSI	0.76 (0.68–0.84)	9.1
STSS	0.75 (0.67–0.82)	1.1
PMEWS	0.72 (0.62–0.80)	8.0
NHAP	0.71 (0.63–0.79)	16.2*
CURB65	0.67 (0.58–0.75)	1.3
CAP	0.66 (0.57–0.75)	1.8

*p<0.05.

**Table 8:** Prediction of ICU admission with fatal cases not admitted to ICU excluded.

Score	ICU Admission AUC (95% CI)	Hosmer-Lemeshow C statistic (Chi-Square)
SMARTCOP	0.74 (0.68–0.79)	33*
MEDS	0.69 (0.63–0.75)	11.0
PSI	0.69 (0.63–0.75)	15.7*
CAP	0.66 (0.60–0.71)	6.9
PMEWS	0.64 (0.58–0.70)	12
STSS	0.64 (0.58–0.71)	2.2
NHAP	0.64 (0.57–0.69)	3.3
CURB65	0.59 (0.53–0.65)	3.0

*p<0.05.

## Discussion

Our study examined the ability of a variety of prognostic scores to predict outcome (death or ICU admission) in a cohort of patients admitted to hospital with laboratory confirmed influenza. Unfortunately, we did not identify any prognostic score with either excellent (AUC>0.90) or good discriminatory ability (i.e. AUC>0.80) for the prediction of either in-hospital mortality or ICU admission.

The score that best predicted in-hospital mortality was the PSI, with an AUC of 0.78. The PSI is a pneumonia severity score developed to assess prognosis in patients with community-acquired pneumonia. It has subsequently been evaluated for use as a tool to help standardize the admission decision for patients with CAP and in one study was demonstrated to reduce the admission of low risk patients with CAP by 18% without adverse consequences [4], [19]. In our cohort, the use of the PSI to identify patients at low risk of in-hospital mortality had the potential to reduce admissions by 35%. The main drawback of the PSI is its complexity. The PSI requires the measurement of 18 variables, including arterial blood gases, 4 additional laboratory variables, and a chest x-ray. However, clinicians' familiarity with this score and its inclusion in widely endorsed CAP guidelines might partially compensate for its complexity. An additional concern is that, although the PSI was a fair predictor of in-hospital mortality, it was a poor predictor of ICU admission. A score that is a poor predictor of the need for ICU admission is not an ideal tool on which to base admission decisions, as patients sent home using such a tool will have a significant risk of requiring readmission and/or experiencing bad outcomes at home.

The MEDS score was also a fair predictor of in-hospital mortality, with an AUC of 0.77 and with 95% confidence intervals similar to those seen with the PSI but with a lower sensitivity than the PSI for the identification of patients at risk of in-hospital mortality (85% vs. 93%). The MEDS score is considerably simpler than the PSI as it requires the measurement of only 7 variables, of which only two are laboratory variables (platelet count and percent bands). The MEDS score also has good face validity for predicting death in influenza patients, as it includes known risk factors for complicated influenza such as age>65, nursing home residence, lower respiratory tract infection, and hypoxemia [20]. In our cohort, the use of the MEDS score in the ER could potentially reduce admissions by 38%. Unfortunately, the MEDS score, like the PSI, was also a poor predictor of the need for ICU admission.

The only score that was a fair predictor of ICU admission was the SMARTCOP score, with an AUC of 0.73. SMARTCOP was also 100% sensitive in identifying patients at risk of ICU admission. However, SMARTCOP had a low specificity (2.8%) for the identification of at-risk patients and therefore would not significantly reduce the number of hospital admissions if used as a triage tool. Furthermore, SMARTCOP was a poor predictor of in-hospital mortality. SMARTCOP is a relatively simple pneumonia severity score comprised of only 8 variables, although it does require arterial blood gases and chest radiography. SMARTCOP was designed to predict which CAP patients will require invasive respiratory or vasopressor support and it is not surprising that it was a better predictor of ICU admission than of in-hospital mortality. The developers of SMARTCOP have commented that the PSI and most other pneumonia severity scores place a considerable emphasis on age and comorbid illnesses as predictors, as opposed to variables focused more on acute physiologic, and specifically respiratory, changes. They also note that older age and severe comorbid illness are predictive of patients being assigned a ‘do not resuscitate’ status; scores that successfully identify patients that die, without excluding patients designated as ‘do not resuscitate’ may also overemphasize the importance of these variables in predicting outcome. When we re-analyze our data after excluding patients that died outside of the ICU, SMARTCOP became the best predictor of in-hospital mortality while remaining the best predictor of ICU admission.

The STSS was a fair predictor of in-hospital mortality, with an AUC of 0.71. The STSS is a sepsis severity score designed to predict in-hospital mortality and the need for critical care resources at the time of assessment in the ER. Furthermore, it was specifically designed as a triage tool for use during influenza epidemics or pandemics, although it has not been previously validated in a cohort of patients with influenza. Unlike the PSI and MEDS, however, the upper 95% confidence interval for the STSS was <0.80 and the specificity for identifying patients at risk of in-hospital mortality was poor at 15%. Thus, our results do not suggest that the STSS will be useful as a tool to guide admission decisions for influenza.

All of the other scores examined performed poorly (AUC<0.70). Of particular note, the other widely used pneumonia severity score, the CURB65 score, was a poor predictor of both in-hospital mortality and ICU admission. This is an important finding, as the CURB65 has been suggested for use as a triage tool for pandemic influenza [7]. PMEWS, another score proposed for use in an influenza pandemic [7], also performed poorly in this cohort.

Of note, we initially considered including acute physiology scores in our analysis (e.g. SOFA, APACHE II, MPMII, etc) however we did not do so as these scores were designed for use in the ICU setting, most of these scores included variables that are only routinely measured in the ICU, and most include levels for each variable that could only occur in ICU patients. For example, the SOFA score assigns a value ranging from 0 to 4 for its ‘respiratory’ variable. Scores >1 require that patients be ventilated. Such scores are unlikely, a priori, to be predictive in the pre-ICU environment, and when they are predictive it is likely that they are predicting the obvious (i.e. an intubated patient in the ER will likely be going to ICU).

Our study has several limitations. Although patients were enrolled prospectively, data collection was retrospective. However, the process of data collection was standardized, the variables included are reasonably objective measures, and there was little missing data. Our cohort is small compared to those used to derive most of the prognostic scores we examined; however, our cohort is large compared to other cohorts of hospitalized patients with laboratory confirmed influenza, and the large sample sizes used to derive scores such as PSI are required in part because of the need to examine large numbers of candidate predictors. Our study was conducted in a single geographical area and the inclusion of only patients with laboratory confirmed influenza and these factors may limit the generalizability of our data to areas with different approaches to influenza screening. Finally, our study examined patients with seasonal, and not pandemic, influenza. Extrapolation to pandemic influenza must be made cautiously. However, a number of groups have suggested that the CURB65, STSS, PMEWS and SOFA scores be used for triage during a pandemic despite the fact that none of these scores has been evaluated in any population with influenza [3], [7]–[11]. Our results are therefore important and should be used to determine which scores should be prioritized for prospective assessment during the current pandemic. Finally, it is possible that patients designated to be at ‘low risk’ for in-patient mortality or ICU admission might have been at low risk only because of supportive therapy they received as inpatients. Therefore, these results cannot be interpreted as predicting outcomes for similar patients in the absence of hospitalization. However, this is true for all pneumonia severity scores; nevertheless, the PSI and CURB65, with careful and ongoing evaluation, have become important management tools for the triage of patients with CAP [4], [6].

In conclusion, our results suggest that currently existent pneumonia severity scores may not be adequately predictive of both in-hospital mortality and the need for ICU admission to be used as decision support tools at the time of initial ER assessment. There is an urgent need to develop such a tool for pandemic influenza, given its potential to prevent a significant proportion of admissions, and to ensure that those patients at risk of severe outcomes are admitted. In our study, only the PSI and MEDS score were moderately predictive of in-hospital mortality. These scores merit further examination for their ability to predict outcome in both seasonal and pandemic influenza. Interestingly, the SMARTCOP score was the best predictor of ICU admission, and the best overall predictor of both outcomes when non-ICU deaths were excluded from the analysis. SMARTCOP also merits further examination, particularly in patients with pandemic influenza, given its relative simplicity and its focus on predicting ICU admission. Future studies should clearly identify patients considered that do not wish to have, or are not considered appropriate for, aggressive care, as these patients should not be included in analyses designed to predict outcomes and intended for use as decision support tools (as such patients would never require ICU care by definition). Our results suggest that the other prognostic scores examined, including the PMEWS, STSS and CURB65, are not sufficiently predictive to be useful as admission tools for seasonal influenza, and raises concern about their use as outcome predictors for pandemic influenza.
